# Supplementary material for: Gene Characterization of Nocturnin Paralogues in Goldfish: Full Coding Sequences, Structure, Phylogeny and Tissue Expression
Source: Int J Mol Sci. 2023 Dec 19;25(1):54. doi: 10.3390/ijms25010054 (PMC10779419; doi:10.3390/ijms25010054)
Supplement: Supplementary file 1 [file ijms-25-00054-s001.zip › ijms-2719042-supplementary.pdf]

Supplementary Table S1. Searching in mRNA SRA libraries of goldfish. Positive hits from BLASTn search of the different splicing variants of *Carassius auratus* nocturnin paralogues obtained from NCBI transcriptomic libraries (SRA).

Nocturnin paralogues	Splicing variants		
	I	II	III
noc-aa	770	1 (0.13%)	2 (0.26%)
noc-ab	899	6 (0.67%)	4 (0.44%)
noc-ba	-	44	-
noc-bb	-	667	3 (0.45%)

SRA accession no: ERX1805732, ERX1805731, SRX668451, SRX3194182, SRX3188220, SRX3185708, SRX3096523, SRX3041132, SRX3041134, SRX1903524, SRX1610992, SRX1167030, SRX999529, SRX824442, SRX668453, SRX177691, SRX176547, DRX013049, SRX383859, SRX375790, SRX316744. Total of 958.1×10^6 sequences.

Supplementary Figure S1. Starting of variants II and III of nocturnins from Cyprinidae

```
                         ┌───>Variant III               Myristoylation
                         │          ┌───>Variant II         score
Cau-AA WNX29031                     MGSSSS---RLFSTVAQTLSSAAL  0.9867
Cau-AA WGS               mESDDML|CQ MGSSSSSS-RLFSTVAQTLGSATL -0.3432
Cgi-AA WGS               MESDDML|CQ MGSSSS---RLFSTLAQTLSSAAL  0.9869
Cca-AA WGS               MKSDDML|CQ MGSSSSS--RLFSTLAQTLSSAAL  0.9871
Sgr-AA WGS               MESDDML|CQ MgSSSSGSSRLFSTLAQTLSSAAL  0.2817

Cau-AB WGS               MESDDML|CQ MGSSSS---RLFSTVAQTLSSAAL  0.9867
Cgi-AB XP_052429539.1 MESDDML|CQ MGSSSSSS-RLFSTVAQTLSSAAL -0.3432
Cca-AB WGS               MESDDML|CQ MGSSS----RLFSTLAQTLSSAAL -0.9849
San-AB WGS               MESDDML|CQ MGSSSSSSSRLFSTLAQTLSNAAL  0.0350
Dre-A  WGS                 MDSDV|CQ MGSNSSS--RLFSTLAQTLSSAAL  0.9861

Cca-BA KTG44155.1            MEVVA|CS MGISTSS---LYSALAKTVSRPPL -0.2097
San-BA XP_016360042.1        MEVAA|CS MGSSTSS---LHSALVKTISRPPL  0.9802

Cau-BB WNX29027              MEVVA|CS MGSSTSS---LYSALAKTISRPAL  0.9788
Cgi-BB XP_052401288.1        MEVVA|CS MGSSTSS---LYSALAKTISRPAL  0.9788
Cca-BB KTF80824.1            MEVVA|CS MGSRTS----LYSALVKTVSRPAL  0.9856
San-BB XP_016347208.1        MEVVA|CS MGSSTSS---LYSALAKTFSRPAL  0.9741
Dre-B  XP_009305064.1        MEVVA|CS MGSGSSS---LYSALAKSISRSPL  0.9855

Xla     MDAQLTYTMGLLEQGYLSARV|CS MGNSTSR---LYSALAKTLSSSAA  0.9891
```

GeneBank accession no. of nocturnin paralogues is indicated for each species: *Carassius auratus* (Cau), *Carassius gibelio* (Cgi), *Cyprinus carpio* (Cca), *Sinocyclocheilus grahami* (Sgr), *Sinocyclocheilus anshuiensis* (San), *Danio rerio* (Dre). *Xenopus laevis* (Xla, AAB39495). When the protein sequence is not available, it has been constructed from Whole-Genome Shotgun contigs database (WGS) with the help of Genewise tool (www.ebi.ac.uk/Tools/psa/genewise/ accessed on 1 September 2023). WGS accession no.: Cau-AA QPKE01002339.1: 1026848-1028993, Cgi-AA JAJQLC010000039.1: 12693683-12695711, Cca-AA JAEOAB010006609.1: c1304546-1302315, Sgr-AA LCYQ01038125.1: c47715-44264, Cau-AB QPKE01000825.1: c289661-288313, Cca-AB JAEOAB010000034.1: c14661701-14659787, San-AB LAVE01186095.1: 4158-6227, Dre-A CABZ01025670.1: 17427-20395. Vertical hyphen indicates the union point between exon 1 and exon 2 in variant III. Lower case letters indicate errors in WGS sequences repaired with the aid of mRNA SRA libraries from the same species. Bold letters indicate the DEG Nend UBRbox 2 motif at the beginning of Variant III, and the myristoylation motif at the beginning of Variant II. The right column includes the scores obtained by Myristoylator tool (https://web.expasy.org/myristoylator/ accessed on 1 September 2023) applied to variant II nocturnins.

Supplementary Figure S2. MTS motifs of Cyprinidae NOC-A Variant I.

Lepisosteus oculatus (XP_006629686.2) (TPpred3 score=0.999) R-3/R-10
MYQSSVRLCSTLLQRDIPVLRVSALGSPVKKAATLKRSSSPGPRYCHSVCADNLSSRTSVPAAPTGLVSLRAVCSMGNSTSRL | Y | SALAQTL | SSTPL

Danio rerio-A (XP_700794.1) (TPpred3 score=0.999) R-10
MYPARRCSSLFHRDLAAVCLSSLGTHPKKSAQPKKNSLQSSRHRSNSSPLLLRRLQVCQMGSNSSSRL | FSTLAQTL | SSAAL

Carassius auratus-AA (WNX29029) (TPpred3 score=0.994) R-10
MYPTRRCSSLLHRELAAVCLSSLGTHPKRHGPLKNSSLPSSRLFQTSVIHPPPTRSNSSPLLLGSLQVCQMGSSSSRL | FSTVAQTL | SSAAL

Carassius auratus-AB (WNX29025) (TPpred3 score=1.0) R-10
MYPARRCSSLFHRDLAAVCLSSLGTHPKKPGPLKKSSLPSSRVHLPPTRSNSSPVLLSSLQVCQMGSSSSRL | FSTVAQTL | SSAAL

Cyprinus carpio-AA (XP_042611323.1) (TPpred3 score=1.0) R-10
MNPARRCSSLLHRELAAVCRSSPGTHPEKPAPLKKSSLPRTRPFHTSVAHPPPTRSSSSPLLLGPLRVCQMGSSSSSRL | FSTLAQTL | SSAAL

Cyprinus carpio-AB (XP_018971399.1) (TPpred3 score=0.999) R-10
MYPARRCSSLLHRDLAAVCLSSLGTHPKKSGPLKKSSFPSSRVHLPPTRSNSSPVLLSSLQVCQMGSSSRL | FSTLAQTL | SSAAL

Sinocyclocheilus anshuiensis-AA-I (XP_016326574.1) (TPpred3 score=0.609) R-10
MYPARRCSSLLHRELAAVCLSLLGTHPKKSGPLKKSSLPSSRLFHTSVVHPPPTRSNSSPALLGSLQVCQMGSSSSGSSRL | FNTLAQTL | SSAAL

Sinocyclocheilus anshuiensis-AB-I (XP_016332140.1) (TPpred3 score=0.999) R-10
MYPARRCSSLLHRDLAAVCLSSLGTHPKKSGPLKKSSLPSSRVHPPPTRSNSSPVLLSSLQVCQMGSSSSSSSRL | FSTLAQTL | SNAAL

Homo sapiens (NP_036250.2) (TPpred3 score=0.997) R-3
MFHSPRRLCSALLQRDAPGLRRLPAPGLRRPLSPPAAVPRPASPRLLAAASAASGAARSCSRTVCSMGTGTSRL | Y | SALAKTLNSSAAS

Xenopus tropicalis (NP_001016531.1) (TPpred3 score=0.717) R-3/R-10
MYQSPASRLSSALRDVLAPCASSLRQHSPVRRRALPTPGQGSLFGLSCCHSLAGWIGEAPGVGHSSSAAAAAAQASATTGTVARSASRTVCSMGNSTSRL | Y | SALAKTL | SSSAA

Underlines indicate predicted α-helix by JPred4 (accessed on 1 September 2023) [48]. Red background indicates basic and cyan hydrophobic amino acids, respectively in initial α-helix. Bold letter indicates consensus residues for mitochondrial peptidases. Mitochondrial Processing Protease (MPP) cleaving site in blue (Rx | [YF]), and octapeptidyl-aminopeptidase (Oct1) cleavage in green ([**FY**]xxxxxxL | S), and Icp55 cleavage in magenta (Y | S). Gar, Human and frog NOC are used as outgroup. For each nocturnin the TPpred3 score (accessed on 1 October 2023, and the principal cleaving motif is included, R-10 (cleavage by MPP + Oct1). For human nocturnin R-3 cleavage (MPP + Icp55) instead of R-10. [51].

Supplementary Table S2. Genome Projects used for synteny analysis.

Species	Assembly	Accession number	Ref.
Danio rerio	GRCz11	GCF_000002035.6	[63]
Carassius auratus	assembly ASM336829v1	GCF_003368295.1	[64]
Carassius gibelio	assembly carGib1.2-hapl.c	GCF_023724105.1	[65]
Cyprinus carpio	assembly ASM1834038v1 (NCBI)<br>Cypcar_WagV4.0 (Ensembl)	GCF_018340385.1<br>GCA_905221575.1	[66]
Sinocyclocheilus grahami	assembly SAMN03320097WGS.v1.1	GCF_001515645.1	[67]

63. Howe, K., Clark, M.D., Torroja, C.F., Torrance, J., Berthelot, C., Muffato, M., Collins, J.E., Humphray, S.', McLaren, K., Matthews, L., McLaren, S., Sealy, I., Caccamo, M., Churcher, C., Scott, C., Barrett, J.C., Koch, R., Rauch, G.J., White, S., Chow, W., Kilian, B., Quintais, L.T., Guerra-Assunção, J.A., Zhou, Y., (153 authors more), Stemple, D.L.. The zebrafish reference genome sequence and its relationship to the human genome. *Nature.* **2013** *496*, 498-503. DOI: 10.1038/nature12111.
64. Chen, Z., Omori, Y., Koren, S., Shirokiya, T., Kuroda, T., Miyamoto, A., Wada, H., Fujiyama, A., Toyoda, A., Zhang, S., Wolfsberg, T.G., Kawakami, K., Phillippy, A.M., Mullikin, J.C., Burgess, S.M. De novo assembly of the goldfish (*Carassius auratus*) genome and the evolution of genes after whole-genome duplication. *Sci Adv*. **2019** *5*, eaav0547. DOI: 10.1126/sciadv.aav0547.
65. Kuhl, H., Du, K., Schartl, M., Kalous, L., Stöck, M., Lamatsch, D.K. Equilibrated evolution of the mixed auto-/allopolyploid haplotype-resolved genome of the invasive hexaploid Prussian carp. *Nat Commun.* **2022** *13,* 4092. DOI: 10.1038/s41467-022-31515-w.
66. Xu, P., Xu, J., Liu, G., Chen, L., Zhou, Z., Peng, W., Jiang, Y., Zhao, Z., Jia, Z., Sun, Y., Wu, Y., Chen, B., Pu, F., Feng, J., Luo, J., Chai, J., Zhang, H., Wang, H., Dong, C., Jiang, W., Sun, X. The allotetraploid origin and asymmetrical genome evolution of the common carp *Cyprinus carpio*. *Nat. Commun*. **2019** *10,* 4625. DOI: 10.1038/s41467-019-12644-1.
67. Yang, J., Chen, X., Bai, J., Fang, D., Qiu, Y., Jiang, W., Yuan, H., Bian, C., Lu, J., He, S., Pan, X., Zhang, Y., Wang, X., You, X., Wang, Y., Sun, Y., Mao, D., Liu, Y., Fan, G., Zhang, H., Chen, X., Zhang, X., Zheng, L., Wang, J., Cheng, L., Chen, J., Ruan, Z., Li, J., Yu, H., Peng, C., Ma, X., Xu, J., He, Y., Xu, Z., Xu, P., Wang, J., Yang, H., Wang, J., Whitten, T., Xu, X., Shi, Q. The *Sinocyclocheilus* cavefish genome provides insights into cave adaptation. *BMC Biol.* **2016** *14*, 1. DOI: 10.1186/s12915-015-0223-4.

Supplementary Table S3. Abbreviations of genes used in the synteny analysis of *nocturnin A*.

Abbreviation	Gene name
anxa5a	*annexin A5a*
bbs7	*Bardet-Biedl syndrome 7*
btbd18	*BTB Domain Containing 18*
c1qtnf2	*C1q and TNF related 2*
cabp2b	*calcium binding protein 2b*
ccdc88b	*girdin-like*
ccg8	*clock controlled gene 8-like*
ccna2	*cyclin-A2-like*
cryba1l1	*beta-crystallin A1-like*
crybb1l1	*beta-crystallin B1-like*
elf2a	*ETS-related transcription factor 2a*
fermt3b	*fermitin family member 3b*
fgfbp1b	*fibroblast growth factor binding protein 1b*
fgfbp2a	*fibroblast growth factor binding protein 2a*
flrt1b	*leucine-rich repeat transmembrane protein 1*
ganab	*neutral alpha-glucosidase AB*
hrasls3	*HRAS-like suppressor 3*
ints5	*integrator complex subunit 5*
ldb2a	*LIM Domain-Binding protein 2*
macrod1	*O-Acetyl-ADP-Ribose Deacetylase 1*
mgarp-like	*mitochondria localized glutamic acid rich protein-like*
mgst2	*microsomal glutathione-S-transferase 2*
naa15a	*N-alpha-acetyltransferase 15 auxiliary subunit-like*
ndufc1	*NADH dehydrogenase [ubiquinone] 1 subunit C1, mitochondrial-like*
noc-a<br>*noc-aa*<br>*noc-ab*	*nocturnin-a (nocturnin-aa, nocturnin-ab)*
prom1a	*prominin 1a*
prpf39	*pre-mRNA-processing factor 39-like*
rab33b	*ras-related protein Rab-33B-like*
rarres3	*retinoic acid receptor responder 3*
rom1b	*RDS/peripherin-like protein xRDS35*
scyl1	*N-terminal kinase-like protein*
setd7	*histone-lysine N-methyltransferase SETD7-like*
slu7	*SLU7 homolog, splicing factor*
tapt1a	*transmembrane anterior posterior transformation protein 1 homolog*
tmem33	*transmembrane protein 33-like*
ubxn1	*UBX domain-containing protein 1*
ugl	*ureidoglycolate lyase*
zgc:113425	*zgc:113425*
**unk**	**unk**nown or uncharacterized gene

Supplementary Table S4. Abbreviations of genes used in the synteny analysis of *nocturnin B*.

Abbreviation	Gene name
ank2a	***ank**yrin **2a**, neuronal*
b4galt1	***b**eta-1,**4**-**gal**actosyl**t**ransferase **1**-like*
c4orf33	*UPF0462 protein **C4orf33** homolog*
camk2d2	***ca**lcium/cal**m**odulin-dependent protein **k**inase type **II** **d**elta **2** chain-like*
chic2	***c**ysteine-rich **h**ydrophobic domain **2***
clta	***cl**a**t**hrin light chain **A**-like*
cplx2l	***c**om**pl**e**x**in **2**-**l**ike*
crmp1	***c**ollapsin **r**esponse **m**ediator **p**rotein **1***
cxcl8a	*chemokine (**C-X-C** motif) **l**igand **8a***
drd1	***d**opamine **r**eceptor **D1***
elf2b	***E**TS-re**l**ated transcription **f**actor **2b***
fip1l1b	*pre-mRNA 3'-end-processing factor **FIP1-l**ike*
gne	*bifunctional UDP-**N**-acetyl**g**lucosamine 2-**e**pimerase/ N-acetylmannosamine kinase-like*
hpf1	***h**istone **P**ARylation **f**actor **1***
mgarpb	***m**itochondria localized **g**lutamic **a**cid **r**ich **p**rotein **b***
nansa	***N-a**cetyl**n**euraminic acid **s**ynthase **a***
noc-b<br>***noc-ba***<br>***noc-bb***	***noc**turnin-**b**, (**noc**turnin-**ba**, **noc**turnin-**bb**)*
ociad1	***OCIA** **d**omain-containing protein **1**-like*
pcdh10a	***p**roto**c**a**dh**erin-**10**-like*
pcdh18a	***p**roto**c**a**dh**erin-**18**-like*
pde5aa	*cGMP-specific 3',**5**'-cyclic **p**hospho**d**i**e**sterase-like*
pgbd3	***p**i**g**gy**B**ac transposable element-**d**erived protein **3**-like*
pgbd4	***p**i**g**gy**B**ac transposable element-**d**erived protein **4**-like*
ppp2r2ca	*serine/threonine-**p**rotein **p**hos**p**hatase **2A** **r**egulatory subunit B*
rbpja	*recombining binding protein suppressor of hairless-like*<br>*= **r**ecombination signal **b**inding **p**rotein for immunoglobulin kappa **J** region **a***
sclt1	***s**odium channel and **cl**a**t**hrin linker **1***
slc34a2a	***s**o**l**ute **c**arrier family **34** member **2a***<br>*= sodium-dependent phosphate transport protein 2B-like*
stim2a	***st**romal **i**nteraction **m**olecule **2**-like*
stra6l	***st**imulated by **r**etinoic **a**cid gene **6** protein-**l**ike*
tcr-like	***T-c**ell **r**eceptor-**like***
tdrd7a	***t**u**d**o**r** **d**omain-containing protein **7A**-like*
tmod1	***t**ropo**mod**ulin-**1**-like*
wfs1a	***W**ol**f**ram **s**yndrome **1a***
zbed4	***z**inc finger **BED**-type containing **4***
zmym1	***Z**inc finger **MYM**-type containing **1***
**unk**	**unk**nown or uncharacterized gene

Supplementary Table S5. Validation of synteny index (SI) and synteny conservation rate (SCR) as diagnostic tools for assigning lineage of orthologous/ohnologous genes.

Golfish paralogue	Orthologous contrast	SI	SCR	Ohnologous contrast	SI	SCR
noc-aa	**Cau-aa/Cgi-aa**	5.5	89.5	**Cau-aa/Cgi-ab**	1.9	65.7
	**Cau-aa/Cca-aa**	8.5	84.6	**Cau-aa/Cca-ab**	2.5	71.4
noc-ab	**Cau-ab/Cgi-ab**	16.5	94.3	**Cau-ab/Cgi-aa**	1.5	60.0
	**Cau-ab/Cca-ab**	4.8	82.9	**Cau-ab/Cca-aa**	3.6	78.4
noc-ba	**Cau-ba/Cgi-ba**	10	90.9	**Cau-ba/Cgi-bb**	0.9	48.1
	**Cau-ba/Cca-ba**	7.2	87.9	**Cau-ba/Cca-bb**	1.7	62.5
noc-bb	**Cau-bb/Cgi-bb**	4.6	82.1	**Cau-bb/Cgi-ba**	1.4	58.1
	**Cau-bb/Cca-bb**	5.6	84.8	**Cau-bb/Cca-ba**	1.4	58.1

Orthologous contrast indicates the comparison of a goldfish *noc* gene with the presumed orthologous in other cyprinine species (comparison matrilineal-matrilineal or patrilineal-patrilineal). Ohnologous contrast indicates the comparison of a goldfish *noc* gene with the presumed ohnologous in other cyprinine species (comparison matrilineal-patrilineal or patrilineal-matrilineal). Cau, *Carassius auratus*; Cgi, *Carassius gibelio;* Cca, *Cyprinus carpio*. SI, synteny index, SCR, synteny conservation rate. Red and blue names indicate matrilineal of patrilineal paralogues.

```
A. ocellaris-A        MGGGSSSSSRLFGTLAQSLNST--QPDPYSEESPN-------------QDLD----HELD 41
S. salar-A            MGSSSSS-SRLFGTLAQTLNSAPLAQQDYDPEHQD-------------SDQDPEGLEQAD 46
S. anshuiensis-AB     MGSSSSSSSRLFSTLAQTLSN--AALADPHVDTDD-------------YEYE-----QAD 40
S. rhinocerous-AB     MGSSSS--SRLFSTLAQTLSS--AALADPHVDTDD-------------YEYE-----QAD 38
C. carpio-AB          MGSSSR----LFSTLAQTLSS--AALADPHVDTDD-------------YEYE-----QAD 36
C. auratus-AB         MGSSSS---RLFSTVAQTLSS--AALTDLHVDTDD-------------DEYE-----QAD 37
S. grahami-AA         MGSSSSGSSRLFSTLAQTLSS--AALEDSHVDSDD-------------YEYE-----QAD 40
S. anshuiensis-AA     MGSSSSGSSRLFNTLAQTLSS--AALADSHVDSDD-------------YEYE-----QAD 40
C. carpio-AA          MGSSSSS--RLFSTLAQTLSS--AALTDPHVDADD-------------YEYE-----QAD 38
C. auratus-AA         MGSSSS---RLFSTVAQTLSS--AALTDPHVDADD-------------HEHE-----QAD 37
D. rerio-A            MGSNSSS--RLFSTLAQTLSS--AALTDPHVDADD-------------YEYE-----QAD 38
I. punctatus-A        MGSSS---VRFFSSLAQSLSS--APLEHTHTDAEE-------------CEPE-----PVD 37
P. nattereri-A        MGSSSS--SRLFSTLAQSLSS--APLAHTHVDPEE-------------CEFE-----QAD 38
C. harengus-A         MGSGSSR-SRLFGTLAQTLSSPSIPLELPALEAEE-------------CACE-----PAD 41
A. ocellaris-B        MGGGAT---RLYRTLTQTLSSTGSPLPLPPSHLDPHQPANTNLDPDFCQEQTGSPLCPPD 57
S. salar-B            MGSGAS---RLYSSLAQTLVNNSTTTTPLAHHQDPQHPS---------QGCHSPPEHPTD 48
S. anshuiensis-BB     MGSSTS---SLYSALAKTFSRPALPHPCDSTYNP--------------GN--DLDQDPAD 41
S. rhinocerous-BB     MGSSTS---SLYSALAKTVSRPALPHPCDSTYNP--------------GNDLDLDQDPAD 43
C. carpio-BB          MGSRTS---GLYSALVKTVSRPALPHPCDSTYNP--------------GNDLDLDQDPAD 43
C. auratus-BB         MGSSTS---SLYSALAKTISRPALPHPCDSTYSP--------------DTNLDLDQDPVD 43
S. anshuiensis-BA     MGSSTS---SLHSALVKTISRPPLPHPCDSTYSP--------------GNDLDLDQDPAD 43
S. rhinocerous-BA     MGSSTS---SLHGALVKTVSRPPLPHPCDSTYSP--------------GNDLDLDQDPAD 43
C. carpioBA           MGISTS---SLYSALAKTVSRPPLPHPCDSTYSP--------------GSDLDLDQNPAD 43
C. auratus-BA         MGISTS---SVFSALAKTISRPPLPHTCD----------------------DLDQNLAD 34
D. rerio-B            MGSGSS---SLYSALAKSISRSPLPHSCGSTYSP--------------GDNLNLDQDPAD 43
C. harengus-B         MGNSPN---RLYSALAPTLSSAPLSQHIPAKLDQ------------------EQDLDPAD 39
L. oculatus           MGNSTS---RLYSALAQTLSSTPLS-QQEQYR----------------EQP--LEVEPID 38
H. sapiens            MGTGTS---RLYSALAKTLNSSAAS-QHPEYL----------------VSPDPEHLEPID 40
X. tropicalis         MGNSTS---RLYSALAKTLSSSAAVSQHQELL----------------ETPDHDQSESLD 41
L. chalumnae          MGNSTS---RLYSALAQTLSSSPLT--PQECA----------------EEPDKEPEEPID 39
                      **  .     .. ::. :.                                        *

A. ocellaris-A        PDQLLRECEEALQRRPARPHRDLVYPSGTEP------SRHKHN------PSIRVMQWNIL 89
S. salar-A            PDQLLKECEEILQNRPARPHRDLVYPADPKH------QHQYRNNEQQQTPSIRVMTWNIL 100
S. anshuiensis-AB     PDALLRECEEVLRNRPPRLHRDFMMTR----------ACSLQN------APIRVMQWNIL 84
S. rhinocerous-AB     PDALLRECEEVLRNRPPRLHRDFMTTR----------ACSLQN------APIRVMQWNIL 82
C. carpio-AB          PDALLRECEDVLRNRPPRLHRDFVMTR----------ACSLQN------APIRVMQWNIL 80
C. auratus-AB         RDALLRECEDVLRNRPPRLHRDFMMTG----------ACSLQS------APIRVMQWNIL 81
S. grahami-AA         PDALLRECEAVLKNRPPRMHRDFVMTR----------ACSLQN------TPIRIMQWNIL 84
S. anshuiensis-AA     PDALLRECEEVLKNRPPRMHRDFVMTR----------ACSLQN------TPIRIMQWNIL 84
C. carpio-AA          PDALLRECEEVLRNRPPRMHRDFVTTR----------AGSLQN------TPIRVMQWNIL 82
C. auratus-AA         PDALLRECEEVLRNRPPRMHRDFVRTR----------ACSLQN------TPIRIMQWNIL 81
D. rerio-A            PDVLLRECGEVLRNRPPRLHREFIMTR----------ACSLQN------SPLRIMQWNIL 82
I. punctatus-A        PETLLRQCEEVLRKRPPRPNREFIRTR----------TSATHD------PQIRVMQWNIL 81
P. nattereri-A        PEALLRECEEVLRNRPPRPHRDFIRTR----------GSTPHN------PQIRVMQWNIL 82
C. harengus-A         PDLLLRECEEALRNRPARWRRRFMRPHTQA-------QAHLDQ------QPIRVMQWNIL 88
A. ocellaris-B        PVELLRRCQEALRDRPPRFHRKLICLSDG--------DGASSS-------PIRVMQWNIL 102
S. salar-B            PLELLRECEEALRDRPPRLLRAFICPGEGEVDREEVDNSATQR-------TIRVMQWNIL 101
S. anshuiensis-BB     PLELLRECREVLRNRPAQLQRAFV-QTGHG--------DARQ--------TIRVMQWNVL 84
S. rhinocerous-BB     PLELLRECREVLRNRPAQLQRAFV-QTGHG--------DARQ--------TIRVMQWNVL 86
C. carpio-BB          PLKLLQECREVLRNRPAQLQRAFI-QTGHG--------DARQ--------TIRVMQWNVL 86
C. auratus-BB         PSELLRECREVLRNRPAQLQRAFV-QTGHG--------DARQ--------TIRVIQWNVL 86
S. anshuiensis-BA     PLELLQECREVLRNRPAQLQRAFV-QTGHG--------DARQ--------TIRVMQWNVL 86
S. rhinocerous-BA     PLELLQECREVLRNRPAQLQRAFV-QTGHG--------DTRQ--------TIRVMQWNVL 86
C. carpioBA           PLELLQECREVLRNRPAQLQRAFV-QTGHG--------DGRQ--------TIRVMQWNVL 86
C. auratus-BA         PVELLQ------------IQRAFL-QTGHG--------YARH--------TIRVMQWNVL 65
D. rerio-B            PLELLQECREALRERPAHLKRAFV-QTGHG--------DARR--------TIRVMQWNVL 86
C. harengus-B         PVELLRECEEVLRTRPARLHRAFVCQNGSG--------DHRQQ-------PIRVMQWNIL 84
L. oculatus           PEELLKKCEEVLQNRPPRLNRSFVFPKKS--------RPSPHR-------PIRVMQWNIL 83
H. sapiens            PKELLEECRAVLHTRPPRFQRDFVDLRTD--------CPSTHP-------PIRVMQWNIL 85
X. tropicalis         PKDLLEECQVALQDRPARLHRDLVSLRND--------SGSQPR-------SFRVMQWNIL 86
L. chalumnae          PDSLLKECREVLRNRPPRFQRDLVNVRAC--------APGGPH-------SMRVMQWNIL 84
                         **.     *:                                      :*:: **:*

A. ocellaris-A        AQALGEGKDGFIRCPLDALNWHERKYLILEEILTYRPDIVCLQEVDHYYDTFLPIMTSLG 149
S. salar-A            AQALGEGKDGFVQCPLDALNWAERKYLILEEILTYRPDILCLQEVDHYYDTFQPILASLG 160
S. anshuiensis-AB     AQALGEGKDGFVRCPMEALNWSERKYLILEEILTYRPDVLCLQEVDHYFDTFQPVLASLG 144
S. rhinocerous-AB     AQALGEGKDGFVRCPMEALNWSERKYLILEEILTYRPDVLCLQEVDHYFDTFQPVLASLG 142
C. carpio-AB          AQALGEGKDGFVRCPMEALNWSERKYLILEEILTYRPDVLCLQEVDHYFDTFQPVLASLG 140
C. auratus-AB         AQALGEGKDGFVRCPMEALNWSERKYLILEEILTYKPDVLCLQEVDHYFDTFQPVLASLG 141
S. grahami-AA         AQALGEGKDGFVRCPMEALNWSERKYLILEEILTYRPDVLCLQEVDHYFDTFRPVLASLG 144
S. anshuiensis-AA     AQALGEGKDGFVRCPIEALNWSERKYLILEEILTYRPDVLCLQEVDHYFDTFQPVLASLG 144
C. carpio-AA          AQALGEGKDSFVRCPMEALNWSERKYLILEEILTYRPDVLCLQEVDHYFDTFQPVLASLG 142
C. auratus-AA         AQALGEGKDGFVRCPMEALNWSERKYLILEEILTYRPDVLCLQEVDHYFDTFQPVLASLG 141
D. rerio-A            AQALGEGKDGFVRCPMEALNWSERKYLILEEILTYRPDVVCLQEVDHYFDTFQPVLSSLG 142
I. punctatus-A        AQALGEGKDGFVRCPMEALVWSERKYLILEEILTYRPDIVCLQEVDHYYDTFQPVMASLG 141
P. nattereri-A        AQALGEGKDGFVRCPMEALNWAERKYLILEEILTYRPDILCLQEVDHYYDTFQPVLSRLG 142
C. harengus-A         AQALGEGKDGFVRCPPEALNWAERKYLILEEILTYQPDILCLQEVDHYYDTFQPVLASLG 148
A. ocellaris-B        AQALGEGLDSFVQCPLEALSWSRRKYLILEEILTYRPHILCLQEVDHYYDTFQPVLAGLG 162
S. salar-B            AQALGEGMDSFVNCPLEALNWAERKYLILEEILTYRPHILCLQEVDHYYDTFQPILASLG 161
S. anshuiensis-BB     AQALGEGMDSFVQCPMDALNWAERKYLILQEILSYRPDIICLQEVDHYFDMFQPVLASLG 144
S. rhinocerous-BB     AQALGEGMDSFVQCPMDALNWAERKYLILQEILTYRPDIICLQEVDHYFDMFQPVLASLG 146
C. carpio-BB          AQALGEGMDSFVQCPMDALNWAERKYLILQEILTYKPDIICLQEVDHYFDMFQPVLASLG 146
C. auratus-BB         AQALGEGMDSFVQCPMDALNWAERKYLILQEIITYRPDIICLQEVDHYFDMFQPVLASLG 146
S. anshuiensis-BA     AQALGEGMDSFVQCPMDALNWAERKYLILQEILTYRPDIICLQEVDHYFDMFQPILASLG 146
S. rhinocerous-BA     AQALGEGMDSFVQCPMDALNWAERKYLILQEILTYRPDIICLQEVDHYFDMFQPILASLG 146
C. carpioBA           AQALGEGMDSFVQCPMDALNWAERKYLILQEILTYRPDIICLQEVDHYFDMFQPILASLG 146
C. auratus-BA         AQALGXGMDSIVQCPMDVLNWAESKYLILQENLTYRPDIICLQEVDQYFDMFQPIMASLG 125
D. rerio-B            AQALGEGMDNFVQCPLDALNWPERKYMILEEILTYKPDILCLQEVDHYFDMFQPVLATLG 146
C. harengus-B         AQALGEGMDSFVRCPLEALKWNERKYLILEEILTYQPDILCLQEVDHYFDTFRPVLSRLG 144
L. oculatus           AQALGEGKDSFVQCPLDALKWAERKYLILEEILTYRPDIFCLQEVDHYFDTFQPIFTSLG 143
H. sapiens            AQALGEGKDNFVQCPVEALKWEERKCLILEEILAYQPDILCLQEVDHYFDTFQPLLSRLG 145
X. tropicalis         AQALGEGKDNFIMCPMEALKWEERKYLILEEILMYQPDVLCLQEVDHYFDTFQPILSRLG 146
L. chalumnae          AQALGEGKDNFILCPMEALNWAERKYLILEEILMHHPDILCLQEVDHYFDTFQPILSRLG 144
                      ***** * *.:: ** :.* * . * :**:* : ::*.:.******:*:* * *::: **
```

```
A. ocellaris-A        YHGSFLAKPWSPCLDVEQNNGPDGCALFYHRSRFSLQDTVHLRLSAMMLPTNQVAIVQTL 209
S. salar-A            YHSTFLPKPWSPCLDVASNNGPDGCALFYRRARFSLLHTSHLRLSAMMLPTNQVAIVQTL 220
S. anshuiensis-AB     YQSSFCPKPCSPCLDVHNNNGPDGCALFFNRRRFQLLHTAHLRLSAMLLKTNQVAIVATL 204
S. rhinocerous-AB     YQSSFCPKPCSPCLDVHNNNGPDGCALFFNRRRFQLLHTAHLRLSAMLLKTNQVAIVATL 202
C. carpio-AB          YQSSFCPKPCSPCLDVHNNNGPDGCALFFNRRRFQLLHTAHLRLSAMLLKTNQVAIVATL 200
C. auratus-AB         YQSSFCPKPCSPCLDVHNNNGPDGCALFFNRRRFQLLHTAHLRLSAMMLKTNQVAIVATL 201
S. grahami-AA         YQSSFCPKPCSPCLDVHNNNGPDGCALFFNRRRFQLLHTAHLRLSVMMLKTNQVAVVATL 204
S. anshuiensis-AA     YQSSFCPKPCSPCLDVHNNNGPDGCALFFNRRRFQLLHTAHLRLSVMMLKTNQVAVVATL 204
C. carpio-AA          YQSSFCPKPCSPCLDVHNNNGPDGCALFFNRRRFQLLHTTHLRLSVMMLKTNQVAVVATL 202
C. auratus-AA         YQSSFCPKPCSPCLDVHNNNGPDGCALFFNRRRFQLLHTTHLRLSVMMLKTNQVAVVATL 201
D. rerio-A            YQSSFCPKPCSPCLDVHNNNGPDGCALFFNRRRFQMLHTAHLRLSAMMLKTNQVAVVATL 202
I. punctatus-A        YQSSFCPKPCSPCLDVSGNNGPDGCALFFSRERFRLLHINHLRLSAMALKTNQVAVVATL 201
P. nattereri-A        YQSTFCPKPCSPCLDVRDNNGPDGCALFFSRQRFQLLCTDHLRLSAMMLKTNQVAIVATL 202
C. harengus-A         YQGSFCPKPWSPCLDVVNNNGPDGCALFFRRDRFEMLSTSHLRLSAMMLKTNQVAVVSTL 208
A. ocellaris-B        YSSHFCPKPWSPCLDVEGNNGPDGCALFFDQSRFELLDSVNIRLSAIRIPTNQVAVVTTL 222
S. salar-B            YRGNFCPKPWSPCLDVEGNNGPDGCALFYDEARFDLVDSVNVRLCAVLIPTNQVAVVTTL 221
S. anshuiensis-BB     YQSSFCPKPWSPCLDVENNNGPDGCALFFNHKCFQSLNTTHLRLSAMMLKTNQVAIITAL 204
S. rhinocerous-BB     YQSSFCPKPWSPCLDVENNNGPDGCALFFNHKRFQSLNTTHLRLSAMMLKTNQVAIITAL 206
C. carpio-BB          YQSSFCPKPWSPCLDVENNNGPDGCALFFNHKRFQSLNTTHLRLSAMMLKTNQVAIIAAL 206
C. auratus-BB         YQSSFYPKPWSPCLDLENNNGPDGCALFFNHKRFQLLNTTHLRLSAMMLKTNQVAIIAAL 206
S. anshuiensis-BA     YQSSFCPKPWSPCLDVENNNGPDGCALFFNPKRFQSLNTTHLRLSAMMLKTNQVAIIAAL 206
S. rhinocerous-BA     YQSSFCPKPWSPCLDVENNNGPDGCALFFNPKRFQSLNTTHLRLSAMMLKTNQVAIIAAL 206
C. carpioBA           YQSSFCPKPWSPCLDVENNNGPDGCALFFNHKRFQSLNTTHLRLSAMMLKTNQVAIIAAL 206
C. auratus-BA         YQSSFCPKPWSPCLEVENKNG-----LFFNHKHFQSLNATHPCLSAMML-TNQVAVIAAL 179
D. rerio-B            YQSSFCPKPWSPCLDVENNNGPDGCALFFNHKRFQLVNTTHLRLSAMMLKTNQVAIVAAL 206
C. harengus-B         YHSSFCPKPWSACLDVANNNGPDGCALFFRHKRFQLLGTTHLQLAAKMLETNQVAIVSTL 204
L. oculatus           YHSSFYPKPWSPCLDVEHNNGPDGCALFFNKERFELVDSTNMRLVAMMLKTNQVAIAQTL 203
H. sapiens            YQGTFFPKPWSPCLDVEHNNGPDGCALFFLQNRFKLVNSANIRLTAMTLKTNQVAIAQTL 205
X. tropicalis         YQCTFLAKPWSPCLDVEHNNGPDGCALFFLQDRFRLVNSAKIRLSARTLKTNQVAIAETL 206
L. chalumnae          YHCSFLPKPWSPCLDVECNNGPDGCALFFNRDKFHLIDSANFRLTVMMLKTNQVAIAQTL 204
                      *   * .** *.**::  :**     **:    *      :  * .  : . ***:  :*

A. ocellaris-A        NCQVTGRWLCVAVTHLKARSGWERLRSAQGADLLQSLRSITSRGSGQ------SEAASG- 262
S. salar-A            RCRETGQRLCVAVTHLKARSGWERLRGAQGADLLQSLKAITSRAAGS------DPASRGP 274
S. anshuiensis-AB     RCRFTGRVFCVAVTHLKARSGWEAFRSAQGAHLLQQLCELTTQSHPE-----VDDENRTR 259
S. rhinocerous-AB     RCRFTGRVFCVAVTHLKARSGWEAFRSTQGAHLLQQLCELTTQSHPE-----MDDENRTR 257
C. carpio-AB          RCRFTGRVFCVAVTHLKARSGWEAFRSAQGAHLLQQLCELTTQSHPE-----MDDENLTR 255
C. auratus-AB         RCRFTGRVFCVAVTHLKARSGWEAFRSAQGAHLLQQLCELTTQSHTE-----MDDENRTR 256
S. grahami-AA         RCRLTGQVFCVAVTHLKARSGWEAFRSAQGAHLLQQLCDLTTQSHPE-----MDDENRTR 259
S. anshuiensis-AA     RCRLTGQVFCVAVTHLKARSGWEAFRSAQGAHLLQQLCDLTTQSHPE-----MDDENRTR 259
C. carpio-AA          RCRLTGQVFCVAVTHLKARSGWEAFRSAQGAHLLQQLCDLTTQSHTE-----MDDENRTR 257
C. auratus-AA         RCRLTGQVFCVAVTHLKARSGWESFRSAQGAHLLQQLCDLTTQSHTE-----MDDENRTR 256
D. rerio-A            RCKLTGRVFCVAVTHLKARSGWEAFRSAQGANLLQQLHEITSQSNPE-----MHQDDQT- 256
I. punctatus-A        QCRVTGRVFCVAVTHLKARSGWETFRGAQGANLLQQLKAILSAQ---------------- 245
P. nattereri-A        LDRVTGRTFCVAVTHLKARSGWEAFRSAQGSHLLQQLRTISSQQGV-------------- 248
C. harengus-A         RCRETGRRFCVAVTHLKARSGWESLRSAQGRSLLQSLGNITMETDPRDAVARGGEADRRR 268
A. ocellaris-B        RCRTTGRCLCVAVTHLKARSGWEWLRSAQGSDLLRHLQNLVQKHS--------------- 267
S. salar-B            RCRVTGRRLCVAVTHLKARSGWERLRSAQGSDLLRNLTTLTQSPGG-------------- 267
S. anshuiensis-BB     RCRATERAFCVAVTHLKARSGWEVLRSAQGSDLLRSLRNVAQSI---------------- 248
S. rhinocerous-BB     RCRATERAFCVAVTHLKARSGWEVLRSAQGSDLLRNLRNVAQRI---------------- 250
C. carpio-BB          RCRATERVFCVTVTHLKARSGWEVLRSAQGSDLLRNLRNVAQRI---------------- 250
C. auratus-BB         RCQATERVFCVAVTHLKARSGWEVLRSAQGSDLLRNLRNVAQRI---------------- 250
S. anshuiensis-BA     RCQATGRAFCVTVTHLKAQSGWEVLRSAQGSDLLRNLRSVVHRI---------------- 250
S. rhinocerous-BA     RCQATGRAFCVTVTHLKARSGWEVLRSAQGSDLLRNLRSVAHRI---------------- 250
C. carpioBA           RCQATGRAFCITVTHLKARSGWEVLRSAQGSDLLRNLRNVTHRI---------------- 250
C. auratus-BA         CCQATGQAFSVTVELLKARSSLEVLRSVQGSDLLQNLRNIAHRI---------------- 223
D. rerio-B            RCRSTGRVFCVGVTHLKARSGWEVLRSAQGSDLLRNLRNITQKI---------------- 250
C. harengus-B         RCRATGRALCVAVTHLKARSGWERLRSAQGSDLLRNLQNITQEAGRAAAAA--------- 255
L. oculatus           RCKETGMLFCVAVTHLKARSGWERFRCSQGSDLLQNLQSITKGTG--------------- 248
H. sapiens            ECKESGRQFCIAVTHLKARTGWERFRSAQGCDLLQNLQNITQGAK--------------- 250
X. tropicalis         QCCETGRLLCFAVTHLKARTGWERFRLAQGSDLLHNLESITEGAT--------------- 251
L. chalumnae          RCKETGKIFCVAVTHLKARSGWERFRFAQGCDLLQNLKNITAGAN--------------- 249
                        :   :.. *  ***::. * :*  **  **: *  :

A. ocellaris-A        ----------------AVPLVVCGDFNAEPSEDVYRRFSSSPLGLSSAYKLLSSDGQTEP 306
S. salar-A            GGG---------SGTEGVPLIVCGDFNAEPSEDVYRRFMSSPLGLDSAYKLLSADGQTEP 325
S. anshuiensis-AB     VGE----------TEDGIPLVVCGDFNAEPSEEVYRRFMTSPLGLDSAYKYLSEDGNTEP 309
S. rhinocerous-AB     VGE----------TEDGIPLVVCGDFNAEPSEEVYRRFMTSPLGLDSAYKCLSEDGNTEP 307
C. carpio-AB          VGE----------TEDGIPLVVCGDFNAEPSEEVYRRFMTSPLGLDSAYKCLSEDGNTEP 305
C. auratus-AB         VGE----------TKDGIPLVVCGDFNAEPSEEVYRRFMTSPLGLDSAYKCLSEDGNTEP 306
S. grahami-AA         VGE----------SEEGIPLVVCGDFNAEPSEEVYRCFMTSPLGLDSAYKCLSEDRNTEP 309
S. anshuiensis-AA     VGE----------SEEGIPLVVCGDFNAEPSEEVYRCFMTSPLGLDSAYKCLSENRNTEP 309
C. carpio-AA          VGE----------SEEGIPLVVCGDFNAEPSEEVYRRFMTSPLGLDSAYKCLSEDGNTEP 307
C. auratus-AA         VWE----------SEEGIPLVVCGDFNAEPSEEVYRRFLSSPLGLDSAYKCLSEDGNTEP 306
D. rerio-A            ---------------EGIPLIVCGDFNAEPNEEVYRHFRSSSLGLDSVYKCLSDDRTTEP 301
I. punctatus-A        -------------QDENVPLFVCGDFNAEPGEEVYRRFMNSPLGLDSAYRTLSS---AEP 289
P. nattereri-A        -------------QPEGVPMVVCGDFNAEPCEEVYRRFLASPLGLDSAYRRLSTDGNTEP 295
C. harengus-A         VGNGLVVQEEGVADEAGVPLIVCGDFNAEPWEDVYKRFASSPLGLDSAYKRLSADGRTEP 328
A. ocellaris-B        -------SGPAGEPKTNIPLLICGDFNAVPSEEVYRRFSTSPLGLDSAYKKLSQDGLTEP 320
S. salar-B            ------PSGPIG-IAPDTPLLVCGDFNAVPSEDVYQRFASSPLILDSAYKRLSRDGLSEP 320
S. anshuiensis-BB     --------ETEENTDTDVPLIVCGDFNAESSEDVYRNFATSSLGLDSAYKHLSADGKTEP 300
S. rhinocerous-BB     --------ETEEKTDTDVPLIVCGDFNAESSEDVYRNFATSSLGLDSAYKHLSADGKTEP 302
C. carpio-BB          --------ETEEKTDTDVPLIVCGDFNAEPSEDVYRNFATSSLGLDSAYKHLSTDGKTEP 302
C. auratus-BB         --------ENEEKTDTDIPLIVCGDFNAEPSEDVYRNFATSSLGLDSAYKHLSADGKTEP 302
S. anshuiensis-BA     --------KTKEKADTDIPLIVCGDFNAEPSEDVYRNFATSSLGLDSAYKRLSTDGKTEP 302
S. rhinocerous-BA     --------KTEEKADSDVPLIVCGDFNAEPSEDIYRNFATSSLGLDSAYKRLSTDGKTEP 302
C. carpioBA           --------KTEEKAETDIPLIVCGDFNAEPSEDVYRNFATSSLGLDSAYKRLSTDGKTEP 302
C. auratus-BA         --------KTEEKADTDIPLIVCGDFNAEPSEDIYRNFATSSLELDSAYKCLSTDGKTEP 275
D. rerio-B            --------ETEENAESAIPLIVCGDFNAEPSEDVYRNFATSSLGLDSAYKLLSTDGKTEP 302
C. harengus-B         ------PGEGEPGVGMGVPLLVCGDFNAEPSEDVYCRFATSSLGLDSAYKRLSSDGQTEP 309
L. oculatus           -----------------IPLLICGDFNAEPTEDVYRHFANSSLNLNSACKLLSHDGQSEP 291
H. sapiens            -----------------IPLIVCGDFNAEPTEEVYKHFASSSLNLNSAYKLLSADGQSEP 293
X. tropicalis         -----------------VPLIICGDFNAEPTEEVYKRFASSSLNLNSAYKLLSEDGESEP 294
L. chalumnae          -----------------VPLIVCGDFNAEPTEEVYRHFASSSLNLNSAYKLLSKDGASEP 292
                                         *:.:****** . *::*  *  *.* *.*. : **    :**
```

```
A. ocellaris-A         AYTTWKIR-PSGESCSTLDYIWYTQEALSVESLLDIPTEEQIGPDRLPSYHYPSDHLSLI 365
S. salar-A             AYTTWKIR-PSGESCSTLDYIWYSHGAFTVDTLLDIPTEEQIGPDRLPSYHYPSDHLSLL 384
S. anshuiensis-AB      PYTSWKIR-PSGESRSTLDYIWYSERAFQVDAVLKIPSEEQIGPDRLPSYHYPSDHLSLV 368
S. rhinocerous-AB      PYTSWKIR-PSGESRSTLDYIWYSERAFQVDTVLKIPSEEQIGPDRLPSYHYPSDHLSLV 366
C. carpio-AB           PYTSWKIR-PSGESRSTLDYIWYSERAFQVDTVLKIPSEEQIGPDRLPSYHYPSDHLSLV 364
C. auratus-AB          PYTSWKIR-PSGESRSTLDYIWYSERAFQVDAVLKIPSEEQIGPDRLPSYHYPSDHLSLV 365
S. grahami-AA          PYTSWKIR-PSGESRSTLDYIWYSERAFQVDTVLRIPNEEQIGPDRLPSYHYPSDHLSLV 368
S. anshuiensis-AA      PYTSWKIR-PSGESRSTLDYIWYSERAFQVDTVLRIPNEEQIGPDRLPSYHYPSDHLSLV 368
C. carpio-AA           PYTSWKIR-PSGESSSTLDYIWYSERAFQVDAVLRIPNEEQIGLNRLPSYHYPSDHLSLV 366
C. auratus-AA          PYTSWKIR-PSGESCSTLDYIWYSERAFQVDAVLRIPNEEQIGPDRLPSYHYPSDHLSLV 365
D. rerio-A             PYTSWKIR-PSGECCSTLDYIWYSEKAFEVDAVLRIPSEEQIGPDRLPSFHYPSDHLSLV 360
I. punctatus-A         SYTTWKIR-PSGESCTTLDYVWFSRHGFSVDGVLSMPSEEQIGPDRLPSYHYPSDHLSLV 348
P. nattereri-A         PYTSWKIR-PSGESCATLDYIWYSRGGFSVDAVLSMPSEEQIGPDRLPSYHYPSDHLSLV 354
C. harengus-A          PYTSWKIRRSSGESCHTLDYIWYSHRRFQVDAVLELPGEEQIGPDRLPSYHYPSDHLSLV 388
A. ocellaris-B         EYTTWKIR-PTGECCSTLDYIWYSQDTLRVDAVLDMPTEEEIGPNRLPSFSYPSDHLSLV 379
S. salar-B             AYTTWKIR-PTGECCTTLDYIWYSREAFRVDAVLDMPTEEQIGPNRLPSYNYPSDHLSLV 379
S. anshuiensis-BB      PYTTWKIR-PSGESCHTLDYVWYSHRAFDVNAVLDFPTAEQIGPNRLPSYNYPSDHLSLV 359
S. rhinocerous-BB      PYTTWKIR-PSGESCHTLDYVWYSHRAFDVNAVLDFPTAEQIGPNRLPSYNYPSDHLSLV 361
C. carpio-BB           PYTTWKIR-PSGESCHTLDYVWYSHRAFDVNAVLDFPTAEQIGPNRLPSYNYPSDHLSLV 361
C. auratus-BB          PYTTWKIR-PSGESCHTLDYVWYSHRAFDVNAVLDFPTAEQIGPNRLPSYNYPSDHLSLV 361
S. anshuiensis-BA      PYTTWKIR-PSGESCHTLDYVWYSHRAFDVNAVLDFPTAEQIGPNRLPSYNYPSDHLSLV 361
S. rhinocerous-BA      PYTTWKIR-PSGESCQTLDYVWYSHRAFDVNAVLDFPTAEQIGPNRLPSYNYPSDHLSLV 361
C. carpioBA            PYTTWKIR-PSGESCHTLDYVWYSHRAFDVKAVLDFPTAEQIGPNRLPSYNYPSDHLSLV 361
C. auratus-BA          PYTTWKIR-PSGESCHTPDY------------------DQIGPNRLPSYNYPSDHLSLV 315
D. rerio-B             PYTTWKIR-PSGESCHTLDYVWYSHRAFDVNAVLDFPTAEQIGPNRLPSYNYPSDHLSLV 361
C. harengus-B          PYTTWKIR-PSGESCHTLDYIWYSHQHLNVDTVLDFPTAEQIGPDRLPSYNYPSDHLSLV 368
L. oculatus            PYTTWKIR-PSGESCHTLDYIWYSQHAFNVDSVLDFPTEEQIGPNRLPSYNYPSDHLSLV 350
H. sapiens             PYTTWKIR-TSGECRHTLDYIWYSKHALNVRSALDLLTEEQIGPNRLPSFNYPSDHLSLV 352
X. tropicalis          PYTTWKIR-PTGESCHTLDYIWYSQHALRVNSALGLPTEEQIGPNRLPSFNYPSDHLSLV 353
L. chalumnae           PYTTWKIR-PTGESCHTLDYIWYSQHALRVDTLLSFPTEDQIGPNRLPSFNYPSDHLSLV 351
                        **:**** .:**.  * **                  ::** :****: ********:

A. ocellaris-A         CDVSFRE---EPHRLI 378
S. salar-A             CDVSFREPRDQPHRLM 400
S. anshuiensis-AB      CDLRFSQQP---HRFM 381
S. rhinocerous-AB      CDLRFSQQP---HRFM 379
C. carpio-AB           CDLSFSQQP---HRFM 377
C. auratus-AB          CDLSFSQKP---HRFM 378
S. grahami-AA          CDLSFSQQP---HRLM 381
S. anshuiensis-AA      CDLSFSQQP---HRLM 381
C. carpio-AA           CDLSFSQQP---HRLM 379
C. auratus-AA          CDLSFSQRP---HRLM 378
D. rerio-A             CDLSFSQQP---HRLM 373
I. punctatus-A         CDFSFIQEP---HRLM 361
P. nattereri-A         CDLSFIQEP---HRLM 367
C. harengus-A          CDFSFSEPP-QTHRLL 403
A. ocellaris-B         CDFSFKEKD------- 388
S. salar-B             CDFSFKKEE---E--- 389
S. anshuiensis-BB      CDFSFKDPA---LVSQ 372
S. rhinocerous-BB      CDFSFKDPM---LVSQ 374
C. carpio-BB           CDFSFKDPA---LVSQ 374
C. auratus-BB          CDFSFKDHT---LVSQ 374
S. anshuiensis-BA      CDFSFKDPT---LVSK 374
S. rhinocerous-BA      CDFSFKDPT---LVSK 374
C. carpioBA            CDFSFKDPT---LVS- 373
C. auratus-BA          CDFSFKDPM---LASK 328
D. rerio-B             CDFCFTDPV---N--- 371
C. harengus-B          CDFSFCDSP---E--- 378
L. oculatus            CDFSFTGEP---DRLL 363
H. sapiens             CDFSFTEES---DGLS 365
X. tropicalis          CDFSFNEDP---ARLL 366
L. chalumnae           CDLSFNEEP---DRLL 364
                       **. *
```

Supplementary Figure S3. Alignment of the deduced amino acid sequences of splicing variant II of nocturnin paralogues from teleosts used to construct phylogenetic trees (Figure 12 and supplementary Figure S4). Multiple sequence alignment was conducted using Clustal X2. Bottom symbols (**. : ***) indicate a significant conservation from minor to major. Species names and GenBank accession numbers of the sequences are as follows: *L. chalumnae*, XP_005996691.1; *X. tropicalis*, KAE8630336.1; *H. sapiens*, NP_036250.2 [truncated]; *L. oculatus*, XP_015200503.1; *C. harengus*-B, XP_012692430.1; *D. rerio*-B, XP_021331689.1; *C. auratus*-BA [Genewise: ID: 113105808], *C. carpio*-BA, XP_018949899.1; *S. rhinocerous*-BA, XP_016375510.1 [truncated]; *S. anshuiensis*-BA, XP_016360043.1; *C. carpio*-BB, XP_018919124.1; *S. rhinocerous*-BB, XP_016410991.1; *S. anshuiensis*-BB, XP_016347209.1; C. auratus-BB, WNX29028; *S. salar*-B, XP_014065414.2; *A. ocellaris*-B, XP_023150319.2; *C. harengus*-A, XP_012689840.1; *D. rerio*-A, XP_005169676.1; *C. carpio*-AA, XP_042625975.1; *S. anshuiensis*-AA, XP_016326574.1 [truncated]; *S. grahami*-AA, XP_016149199.1 [truncated]; *C. auratus*-AA, WNX29031; *C. carpio*-AB, XP_018971399.1 [truncated]; *S. rhinocerous*-AB, XP_016429112.1; *S. anshuiensis*-AB, XP_016332140.1 [truncated]; *C. aurarus*-AB, WNX29026; *P. nattereri*-A, XP_017547499.1; *I. punctatus*-A, XP_017329141.1; *S. salar*-A, XP_014055071.1; *A. ocellaris*-A, XP_035806733.2. For *C. auratus*-BA sequence, pseudogene has been translated to protein with Genewise tool (https://www.ebi.ac.uk/Tools/psa/genewise/ accessed on 1 September 2023).

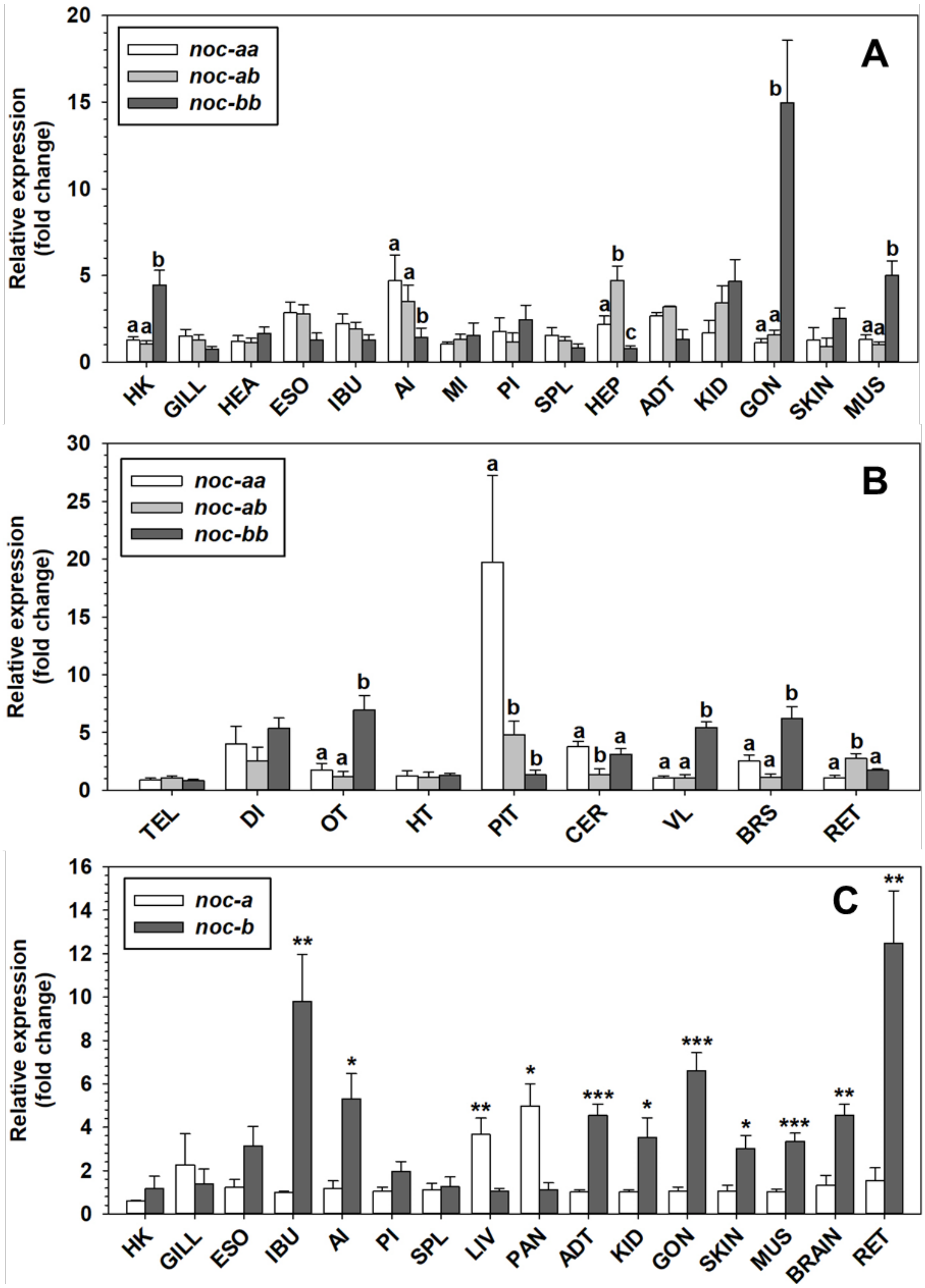

Supplementary Figure S4. Relative abundance of nocturnin paralogues in several tissues. (A) goldfish peripheral tissues, (B) neural locations, and (C) zebrafish tissues. Data are expressed as mean + S.E.M. (n = 5-6) relative to the paralogue with lower expression for each tissue. Different letters in A and B indicate statistical differences among goldfish paralogues (ANOVA-SNK; p<0,05). Asterisks indicate statistical differences between zebrafish paralogues expression (* p<0,05; ** p<0,01; *** p<0,001). Head kidney (HK), gill (GILL), heart (HEA), esophagus (ESO), intestinal bulb (IBU), anterior intestine (AI), middle intestine (MI), posterior intestine (PI), spleen (SPL), hepatopancreas (HEP), liver (LIV), pancreas (PAN), adipose tissue (ADT), caudal kidney (KID), gonad (GON), skin (SKIN), muscle (MUS), telencephalon (TEL), diencephalon (DI), optic tectum (OT), hypothalamus (HT), pituitary (PIT), cerebellum (CER), vagal lobe (VL), brainstem (BRS), brain (BRAIN), retina (RET).

Supplementary Table S6. Primers used for cloning and expression analysis of goldfish and zebrafish *nocturnin* paralogs.

Species	Primer name	Sequence (5´to 3´)	Target gene	Application
Universal	M13 Forward	GTAAAACGACGGCCAG	TOPO vector insertions	Cloning
	M13 Reverse	CAGGAAACAGCTATGAC		
	T3 Forward	ATTAACCCTCACTAAAGGGA		
	T7 Reverse	TAATACGACTCACTATAGGG		
Goldfish	NOCA1 F9	GGGCTGTCAACCGAAGAAGA	*noc-aa*	
	NOCA1 F10	AACTACGGTTCGAAGGGCTG		
	NOCA R6	AGCTGGAAGCGACGTCGG		
	NOCA2 F1	GCTTTCCGAGTTCACGAGTTCATCTTCC	*noc-ab*	
	NOCA2 R2	CCACTAACGGAATACCGTCTTTGG		
	NOCB1 F18	AAGACCATCAGCAGACCTGC	*noc-bb*	
	NOCB1 R7	TGGGTCCAATTTGTTCGGCT		
	NOCA1 QF1	GCTGTGCTGAGGATACCGAA	*noc-aa*	RT-qPCR
	NOCA1 QR1	TACATGAGCCTATGAGGCCG		
	NOCA2 QF2	ATGCTGTGCTAAAGATACCAAG	*noc-ab*	
	NOCA2 QR2	GCTACATGAACCTATGAGGCTT		
	NOCB1 F8	AAGTGGCGAGAGCTGCCAC	*noc-bb*	
	NOCB1 R10	AAGGAAGCCTATTGGGTCC		
	NOCA2 F2	GCTTAGCCATAGTTTGGCTT	*noc-ab* splicing variant I	
	NOCA2 F21	ACTAACCCACTGAACGGACAC	*noc-ab* splicing variant II	
	NOCA2 R14	GGAGGCCGGTTCCTCAAAACA	*noc-ab* splicing variant I and II	
	β-actin Forward	CTACTGGTATTGTGATGGACT	*β-actin* [60]	
	β-actin Reverse	TCCAGACAGAGTATTTGCGCT		
	ef-1α Forward	CCCTGGCCACAGAGATTTCA	*ef-1α* [60]	
	ef-1α Reverse	CAGCCTCGAACTCACCAACA		
Zebrafish	NOCA F	AGCCTTCAGAGTTCACGACA	*noc-a* [5]	
	NOCA R	GGCTCTGGTCATGATGAACT		
	NOCB F	ACAATCTGAATCTGGATCAG	*noc-b* [5]	
	NOCB R	TCCCTCCAGGATCATGTACTT		
	β-actin Forward	ACGAACGACCAACCTAAACCTC	*β-actin* [61]	
	β-actin Reverse	TTAGACAACTACCTCCCTTTCC		
